# Serum Levels of Selected Chemokines in Patients with Systemic Lupus Erythematosus Correlate with Disease Activity and Clinical Features: Results from a Single-Center Observational Study

**DOI:** 10.3390/biomedicines14030567

**Published:** 2026-03-02

**Authors:** Radosław Dziedzic, Andżelika Siwiec-Koźlik, Paweł Koźlik-Siwiec, Kazimierz Węglarczyk, Maciej Siedlar, Mariusz Korkosz, Joanna Kosałka-Węgiel

**Affiliations:** 1 Jagiellonian University Medical College, Doctoral School of Medical and Health Sciences, św. Łazarza 16, 31-530 Kraków, Poland; radoslaw.dziedzic@doctoral.uj.edu.pl; 2 University Hospital in Kraków, Clinical Department of Rheumatology, Immunology and Internal Medicine, Jakubowskiego 2, 30-688 Kraków, Poland; lek.andzelika.siwiec@gmail.com (A.S.-K.); mariusz.korkosz@uj.edu.pl (M.K.); 3 University Hospital in Kraków, Clinical Department of Hematology and Internal Medicine, Jakubowskiego 2, 30-688 Kraków, Poland; pawelkozlik89@gmail.com; 4 Jagiellonian University Medical College, Department of Clinical Immunology, Wielicka 265, 30-663 Kraków, Poland; kazimierz.weglarczyk@uj.edu.pl (K.W.); maciej.siedlar@uj.edu.pl (M.S.); 5 Jagiellonian University Medical College, Department of Rheumatology and Immunology, Jakubowskiego 2, 30-688 Kraków, Poland

**Keywords:** systemic lupus erythematosus, biomarkers, chemokines, CCL2, CCL4, CCL5, CXCL8, CXCL10

## Abstract

**Background/Objectives**: Systemic lupus erythematosus (SLE) is a chronic autoimmune disease characterized by fluctuating disease activity and multi-organ involvement. The identification of reliable biomarkers that accurately reflect disease activity remains a significant clinical challenge, particularly in predicting disease flares. Chemokines are key mediators of immune cell recruitment and inflammation, making them promising candidates for disease activity monitoring. Therefore, we evaluated serum concentrations of CCL2, CCL4, CCL5, CXCL8, and CXCL10 and examined their associations with disease activity and clinical manifestations in patients with SLE. **Patients and Methods**: A total of 52 patients with SLE were enrolled in the study, of whom 15 (28.8%) had active disease (SLE Disease Activity Index [SLEDAI] ≥ 5) and 37 (71.2%) were in remission (SLEDAI < 5). An additional group of 12 age- and sex-matched healthy individuals without a family history of autoimmune diseases served as controls. All SLE patients fulfilled the 2019 EULAR/ACR classification criteria. Serum levels of the selected chemokines were measured in all participants using the Luminex Human Premixed Multi-Analyte Discovery Assay. **Results**: Serum concentrations of CCL2 and CCL4 did not differ between SLE patients and healthy controls, nor between active and inactive disease subgroups (*p* > 0.05, for all). In contrast, CCL5 levels were 34.30% higher in patients with SLE compared with controls (*p* = 0.013), with the strongest increase observed in the inactive disease subgroup as compared to controls (by 40.29%, *p* = 0.021). CXCL8 levels were markedly elevated in patients with active SLE relative to those in remission (by 123.30%, *p* = 0.011) and to healthy individuals (by 183.96%, *p* = 0.049). CXCL10 levels were higher in both active and inactive SLE groups compared with controls (increase of 180.80%, *p* < 0.001 and increase of 100.80%, *p* = 0.018, respectively). No differences in chemokine levels were detected between patients with renal flares and those with non-renal flares, nor among patients in remission with and without a history of lupus nephritis (*p* > 0.05, for all). CXCL8 and CXCL10 correlated positively with disease activity scores, inflammatory markers, and several immune parameters, indicating their relevance to ongoing inflammatory processes (*p* < 0.05, for all). CCL5 was associated with complement components C3 (r_S_ = 0.36, *p* = 0.008) and C4 (r_S_ = 0.38, *p* = 0.006), while CXCL10 showed negative correlations with white blood cell (r_S_ = −0.34, *p* = 0.013), lymphocyte counts (r_S_ = −0.36, *p* = 0.008) and neutrophils (r_S_ = −0.32, *p* = 0.019). In the longitudinal follow-up of patients in remission (median follow-up time of 5.5 years), baseline chemokine levels did not predict subsequent disease flares among SLE patients who were inactive as the study baseline (*p* > 0.05, for all). **Conclusions**: In our study, serum levels of CXCL8 and CXCL10 reflect disease activity and systemic inflammation in SLE, supporting their potential value as biomarkers for monitoring ongoing immune activation. Baseline concentrations of the examined chemokines did not predict future disease flares, indicating their limited utility in this context.

## 1. Introduction

Systemic lupus erythematosus (SLE) is a chronic autoimmune disease characterized by dysregulation of innate and adaptive immune response, autoantibody production, formation of circulating immune complexes, leukocyte activation and recruitment, and subsequent end-organ damage conferring high morbidity and a risk of fatal outcome [1]. The disease is characterized by fluctuating course of flares and remission, with the known risk factors of relapse being patient-related (e.g., younger age at diagnosis, African American descent), disease-related (history of renal involvement, presence of anti-Smith (anti-Sm) or anti-double stranded DNA (anti-dsDNA) antibodies), treatment-related (e.g., the need for low-dose glucocorticosteroids maintenance), and environmental (e.g., ultraviolet light exposure, influenza virus infection) [2,3]. A major challenge in SLE management is the identification of biomarkers that can accurately reflect disease activity and predict future flares [4]. Such a predictive tool could inform maintenance immunosuppressant use, disease monitoring frequency, as well as lifestyle and occupational choices. While anti-dsDNA titer and serum complement(C)3 and C4 concentrations were established as early markers or predictors of disease flares, their clinical utility is limited by suboptimal sensitivity and specificity [5].

Interestingly, chemokines play a central role in the immunopathogenesis of SLE, mediating leukocyte recruitment, activation, and tissue infiltration [6]. Among the vast chemokine network, CCL2 (C-C motif chemokine ligand 2 [MCP-1]), CCL4 (C-C motif chemokine ligand 4 [MIP-1β]), CCL5 (C-C motif chemokine ligand 5 [RANTES]), CXCL8 (C-X-C motif chemokine ligand 8 [IL-8]), and CXCL10 (C-X-C motif chemokine ligand 10 [IP-10]) have been identified as key drivers of the inflammatory cascade [7]. CCL2 (MCP-1) is consistently elevated in SLE and implicated in monocyte recruitment, particularly in lupus nephritis (LN) and neuropsychiatric SLE, where it correlates with end-organ damage [6,8,9]. CCL4 (MIP-1β) and CCL5 (RANTES) are produced by activated T-cells, endothelial cells, and platelets, and act primarily through the CCR5 receptor to recruit T-cells, monocytes, and macrophages to sites of tissue injury. Increased serum and renal expression of CCL5 is associated with active SLE and LN, and experimental models show that RANTES deficiency or blockade attenuates renal inflammation and proteinuria, indicating a direct pathogenic role [10,11]. CXCL8 is produced by monocytes, macrophages, and endothelial cells in response to inflammatory stimuli, and acts via CXCR1 and CXCR2 receptors to promote neutrophil chemotaxis, degranulation, and the formation of neutrophil extracellular traps (NETs), which are implicated in SLE pathogenesis through the provision of autoantigens and perpetuation of tissue injury [11,12]. CXCL10 (IP-10) is induced by interferon-γ (IFN-γ) along with CXCL9 and CXCL11, which share the CXCR3 receptor. It promotes Th1/Th17 polarization as well as recruitment of monocytes, macrophages, T-cells, and NK cells. It is strongly associated with disease activity and acts as a marker for therapeutic response, though high levels may persist in treatment-refractory cases, especially a subset of patients with high STAT1 leukocyte expression [9,13,14]. Despite the clear mechanistic involvement of these mediators [15,16], chemokine testing is not yet standard in clinical practice. While elevated baseline levels of interferon-inducible chemokines (such as CCL2 and CXCL10) have been linked to severe disease and specific autoantibody profiles [17,18,19], there are barriers related to assay standardization, variability among patients, and insufficient evidence of added clinical value [15,16]. Most importantly, the cross-sectional design of most studies lacks follow-up to confirm role in flare prediction [8,18,20]. Hence, further prospective research is required to determine if chemokine profiling can truly improve flare risk stratification [18,19,21].

Therefore, the aim of this single-center, longitudinal observational study was to evaluate serum concentrations of CCL2, CCL4, CCL5, CXCL8, and CXCL10 in patients with SLE compared to healthy controls, examine their associations with current disease activity (SLE Disease Activity Index; SLEDAI) and clinical manifestations to address issues of specificity, and assess the prognostic utility of baseline chemokine levels in predicting disease flares during a longitudinal follow-up period, addressing the paucity of prospective data in the field.

## 2. Patients and Methods

### 2.1. Characteristics of the Patients

The study included 52 individuals with a confirmed diagnosis of SLE based on the 2019 European League Against Rheumatism and the American College of Rheumatology (EULAR/ACR) classification criteria [22]. Demographic and clinical information was collected, including sex, current age, age at disease onset, and total disease duration. Peripheral blood samples were drawn from all participants for the assessment of chemokine concentrations. In patients who had undergone kidney biopsy, the histopathological classification of LN was determined using the International Society of Nephrology/Renal Pathology Society (ISN/RPS) criteria [23,24]. None of the individuals had a history of treatment with B-cell-targeted or IFN-related biologic agents (e.g., rituximab, belimumab, anifrolumab). Medical records were reviewed to determine the dose of systemic glucocorticosteroids (sGCS) at the time of sampling and the cumulative cyclophosphamide (CTX) dosage. Importantly, no CTX therapy was administered during the six months preceding blood collection. Disease activity was evaluated using the SLE Disease Activity Index (SLEDAI) and its renal component (rSLEDAI) [25,26], with active disease defined as a SLEDAI score ≥ 5 [27,28,29]. Next, a disease flare during follow-up was defined as a physician-adjudicated clinical worsening attributable to SLE, accompanied by an increase in SLEDAI score, in total ≥ 5points.

Each patient underwent a comprehensive clinical assessment to document both past and present SLE-related manifestations. Definitions of clinical manifestations were based on current literature on SLE and previous papers [30,31,32]. Briefly, cutaneous involvement included malar erythema, discoid plaques, or other lupus-specific dermatologic lesions. Oral ulcerations were defined as painless mucosal defects typically located on the hard palate, buccal mucosa, or nasal septum. Photosensitivity referred to skin reactions induced or aggravated by sunlight or ultraviolet exposure, potentially associated with disease worsening. Articular involvement was characterized by non-erosive arthritis or joint pain, most commonly affecting small joints of the hands. Serositis was diagnosed based on clinical signs or imaging evidence of pleuritis or pericarditis. Renal involvement included proteinuria, hematuria, or reduced kidney function, with biopsy confirmation in selected cases. Neuropsychiatric manifestations comprised central or peripheral nervous system symptoms such as seizures, psychosis, or cognitive impairment attributable to SLE. Hematological abnormalities included anemia (including autoimmune hemolytic anemia), leukopenia, lymphopenia, or thrombocytopenia, consistent with accepted diagnostic criteria.

The study protocol was reviewed and approved by the Bioethics Committee of the Jagiellonian University Medical College (approval No. 1072.6120.34.2018, dated 23 February 2018). All procedures adhered to the ethical principles of the Declaration of Helsinki. Written informed consent was obtained from every participant prior to study inclusion.

### 2.2. Laboratory Investigations

Laboratory evaluation included hematologic, renal, and immunological markers. Complete blood count (CBC), C-reactive protein (CRP), serum creatinine with estimated glomerular filtration rate (eGFR; calculated using the MDRD equation), 24 h urinary protein excretion, and urine sediment were determined according to routine diagnostic procedures. Anti-nuclear antibodies (ANA) were detected by indirect immunofluorescence (IIF) using HEp-2 cell substrates, in accordance with standard laboratory procedures. Serum samples were initially screened at a dilution of 1:80, and positive samples were further titrated by serial twofold dilutions to determine endpoint titers. ANA patterns were evaluated by experienced laboratory personnel using fluorescence microscopy. Anti–double-stranded DNA (anti-dsDNA) antibodies were assessed by both IIF on *Crithidia luciliae* and enzyme-linked immunosorbent assay (ELISA; EUROIMMUN, Lübeck, Germany). When both ELISA and *Crithidia luciliae* immunofluorescence assays were available, the higher anti-dsDNA antibody result was considered the final value for analysis. Serum complement levels (C3 and C4) were quantified by laser nephelometry. For chemokine assessment, 5 mL of peripheral blood was collected into clot-activator tubes. Samples were centrifuged within two hours of collection at 4000 rpm for 5 min, and the resulting serum was transferred to microtubes and clarified by a second centrifugation cycle at 14,000 rpm for 10 min. Aliquots of serum were then stored at −80 °C until analysis. Chemokine concentrations (CCL2, CCL4, CCL5, CXCL8, and CXCL10) were measured using the Human Premixed Multi-Analyte Luminex Discovery Assay (R&D Systems, Minneapolis, MN, USA), and data acquisition was performed on the Luminex 200 system (Luminex Corporation, Austin, TX, USA) in accordance with the manufacturer’s instructions.

### 2.3. Statistical Elaboration

All statistical procedures were performed using IBM SPSS Statistics software (version 29.0.0.0, IBM Corp., Armonk, NY, USA). Plots and data visualizations were created in GraphPad Prism (version 10.4.0, GraphPad Software, San Diego, CA, USA). Categorical variables were analyzed using the Chi2 test or Fisher’s exact test when appropriate and presented as absolute numbers (*n*) with corresponding percentages. To verify whether the study sample was sufficient (15 patients with active SLE, 37 in remission, and 12 healthy controls), a priori power estimation was conducted. With a presumed large effect size (Cohen’s f = 0.4) and α = 0.05, the resulting statistical power was 0.805 (β = 0.195), meeting the standard criterion of ≥0.80 recommended for biomedical research. Continuous variables did not meet the assumptions of normal distribution; therefore, they are reported as medians with Q1–Q3 ranges. Comparisons between two independent groups were carried out using the Mann-Whitney test. Differences in chemokine concentrations across the three groups (active SLE, inactive SLE and controls) were assessed using the Kruskal-Wallis test followed by Bonferroni-adjusted post hoc comparisons. This same approach was applied to the analysis of demographic and laboratory parameters. Associations between continuous variables were evaluated using Spearman’s rank correlation coefficient (r_S_). Statistical significance was defined as *p* < 0.05.

## 3. Results

### 3.1. Characteristics of the Study Participants

The study included 52 individuals diagnosed with SLE (median age 49.0 years; 88.5% women) and 12 healthy control subjects (median age 37.5 years; 66.7% women). No significant differences were detected between the two groups about major demographic characteristics such as age or sex. Likewise, when comparing patients with active versus inactive SLE, variables including age, sex, age at disease onset, and disease duration did not differ significantly. A detailed summary of the demographic, clinical, and laboratory data is provided in Table 1 (based on our previous papers [31,32]).

Among the SLE-related clinical manifestations present in the cohort, hematologic involvement was the most frequently reported (47 patients, 90.4%), followed by articular symptoms (41 patients, 78.8%) and skin-related lesions (37 patients, 71.2%). Conversely, oral ulcers (5 patients, 9.6%) and neurological complications (3 patients, 5.8%) occurred relatively rarely. All individuals demonstrated ANA positivity at titers ≥ 1:160, while anti-dsDNA antibodies were identified in 24 participants (46.2%), making them the most common disease-specific autoantibody observed. At the time of evaluation, participants with active SLE more often presented with cutaneous manifestations, serositis, and renal or hematologic abnormalities compared with those in remission. LN was diagnosed in 24 patients (46.2%), and 8 of them (33.3%) were experiencing a renal flare at study entry. Clinically, active disease was associated with higher SLEDAI and rSLEDAI scores. These patients additionally exhibited increased ANA titers and elevated anti-dsDNA antibody levels. Complement C4 levels and lymphocyte counts were decreased in those with active SLE, whereas proteinuria was more pronounced. Relative to healthy controls, patients with active SLE showed lower lymphocyte counts, higher CRP concentrations, and increased proteinuria. Three individuals were taking methylprednisolone at daily doses > 8 mg. Furthermore, 23 participants (44.2%) had previously received cyclophosphamide therapy more than six months before sampling, with a mean cumulative dose of 24.2 g. At the time of enrollment, apart from hydroxychloroquine, only two patients were receiving additional immunosuppressive medications, namely azathioprine and mycophenolate mofetil.

### 3.2. Laboratory Measurements of Selected Cytokines

**Figure 1:**
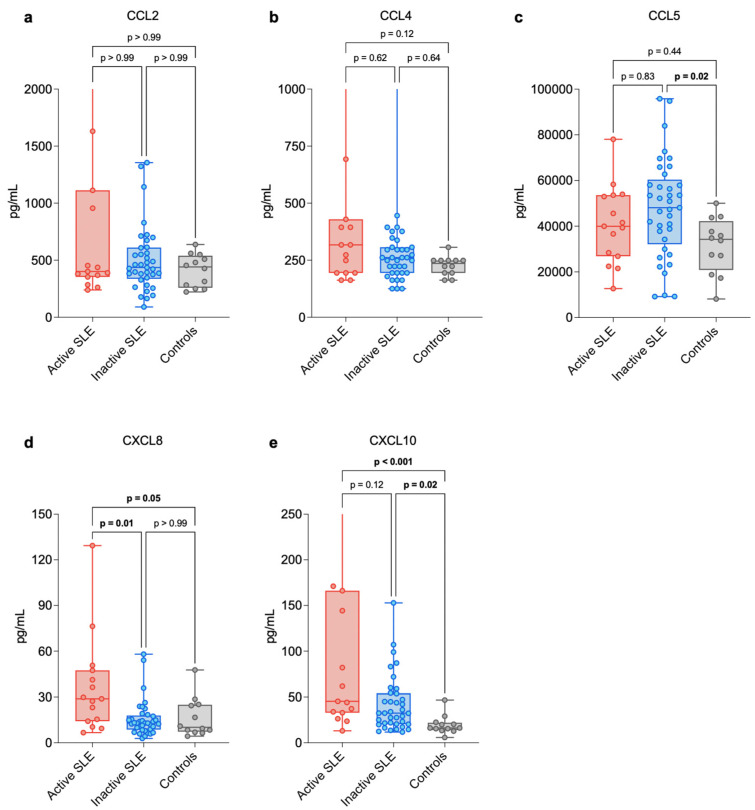
Levels of CCL2 (**a**), CCL4 (**b**), CCL5 (**c**), CXCL8 (**d**) and CXCL10 (**e**) in active (n = 15) and inactive (n = 37) patients with systemic lupus erythematosus and a control group (n = 12). Data are presented as medians with Q1–Q3 ranges and minimum–maximum values. Each dot represents a specific cytokine measurement. Statistically significant differences are bolded. Abbreviations: CCL5—C-C motif chemokine ligand 5 (RANTES), CCL2—C-C motif chemokine ligand 2 (MCP-1), CCL4—C-C motif chemokine ligand 4 (MIP-1β), CXCL10—C-X-C motif chemokine ligand 10 (IP-10), CXCL8—C-X-C motif chemokine ligand 8 (IL-8), SLE—systemic lupus erythematosus. Some extreme values were outside the plotting range and therefore are not visible in the figure; however, they were included in all statistical analyses.

Serum concentrations of selected chemokines were evaluated in all study participants. The complete results are shown in Figure 1a–e and summarized in Table 2. Levels of CCL2 and CCL4 did not differ significantly between SLE patients and healthy controls, nor between active and inactive SLE groups (*p* > 0.05 for all comparisons). In contrast, CCL5 levels were significantly higher in the overall SLE cohort compared with controls (increase of 34.30%, *p* = 0.013), and this difference remained significant specifically in the inactive SLE subgroup (increase of 40.29%, *p* = 0.021). No significant difference was observed between the active SLE subgroup and controls (*p* = 0.44). CXCL8 concentrations were markedly elevated in patients with active SLE when compared to those in remission (increase of 123.30%, *p* = 0.011) and higher relative to controls (increase of 183.96%, *p* = 0.049). The most substantial differences were observed for CXCL10. Its serum levels were significantly increased in the entire SLE group versus healthy controls (increase of 116.72%, *p* < 0.001), in the active SLE subgroup relative to controls (increase of 180.80%, *p* < 0.001), and in inactive SLE when compared with controls (increase of 100.80%, *p* = 0.018).

### 3.3. Detailed Characteristics of Patients with Active Lupus Nephritis and Those with Exacerbated Systemic Lupus Erythematosus Without Active Nephritis

We divided active SLE patients into two subgroups: with renal flare (n = 8) and non-renal flare (n = 7). Nevertheless, there were no differences in levels of selected chemokines (Table 3).

### 3.4. Characteristics of Inactive Systemic Lupus Erythematosus Patients

Next, we divided remissive SLE patients into two subgroups: patients with renal remission (n = 16) and patients in remission with never diagnosed LN (n = 21). However, there were no differences in levels of analyzed chemokines (Table 4).

### 3.5. Serum Levels of Selected Chemokines Correlate with Clinical and Laboratory Parameters of Systemic Lupus Erythematosus

In fact, CCL2 showed a moderate positive association with CXCL8 (r_S_ = 0.48, *p* < 0.001) and a weaker correlation with CXCL10 (r_S_ = 0.28, *p* = 0.048) and CCL4 (r_S_ = 0.28, *p* = 0.045). CCL4 was correlated with CCL2 as described, but also strongly correlated with CXCL8 (r_S_ = 0.51, *p* < 0.001) and CXCL10 (r_S_ = 0.55, *p* < 0.001). CCL5 did not show significant correlations with the remaining chemokines (*p* > 0.05, for all). In contrast, CXCL8 was positively associated not only with CCL2 and CCL4, but also with CXCL10 (r_S_ = 0.41, *p* = 0.002). Likewise, CXCL10 demonstrated significant correlations with CCL2, CCL4, and CXCL8, as described.

The analysis of associations between the measured chemokines and clinical as well as laboratory parameters in the SLE group revealed several significant findings. Details were provided below in Table 5. CXCL8 and CXCL10 showed the strongest relationships with disease activity. CXCL8 levels demonstrated a positive correlation with both the SLEDAI (r_S_ = 0.44, *p* = 0.001) and rSLEDAI scores (r_S_ = 0.42, *p* = 0.002), as well as with proteinuria (r_S_ = 0.29, *p* = 0.040) and C-reactive protein levels (r_S_ = 0.42, *p* = 0.003). CXCL10 levels also correlated positively with overall disease activity (SLEDAI: r_S_ = 0.37, *p* = 0.007) and ANA titers (r_S_ = 0.35, *p* = 0.011), and were associated with higher anti-dsDNA antibody levels (r_S_ = 0.29, *p* = 0.036). Regarding immunological markers, CCL5 was positively associated with both complement C3c (r_S_ = 0.36, *p* = 0.008) and complement C4 (r_S_ = 0.38, *p* = 0.006). Additionally, CCL4 demonstrated an inverse correlation with complement C4 (r_S_ = −0.34, *p* = 0.013). Several chemokines also demonstrated relationships with inflammatory and hematological parameters. CXCL10 and CXCL8 both showed positive correlations with CRP (r_S_ = 0.45, *p* < 0.001 and r_S_ = 0.42, *p* = 0.003, respectively). CXCL10 further exhibited negative correlations with white blood cell count (r_S_ = −0.34, *p* = 0.013), lymphocyte count (r_S_ = −0.36, *p* = 0.008) and neutrophil count (r_S_ = −0.32, *p* = 0.019). CCL4 displayed a negative correlation with current systemic glucocorticoid dose (r_S_ = −0.29, *p* = 0.040). Finally, CCL2 was positively associated with anti-dsDNA antibody levels (r_S_ = 0.38, *p* = 0.005), indicating a potential link between CCL2 expression and autoimmune activity.

### 3.6. Follow-Up Assessment of Patients with Inactive Systemic Lupus Erythematosus

In the longitudinal assessment, we monitored patients who were in remission at study entry over a median follow-up period of 5.5 years (Table 6). During this interval, 10 individuals experienced a disease flare, with the median time to flare being approximately 2 years after enrolment. Baseline concentrations of the analyzed chemokines did not differ between patients who subsequently developed a flare and those who remained in sustained remission.

## 4. Discussion

In this paper, we analyzed serum levels of selected chemokines including CCL2, CCL4, CCL5, CXCL8, and CXCL10 in patients with active and inactive SLE compared with healthy controls. By integrating cross-sectional analysis with a longitudinal follow-up, we aimed to distinguish between biomarkers that reflect concurrent disease activity and those that hold prognostic value for future flares. Our primary findings indicate that while serum CXCL8 and CXCL10 are robust markers of ongoing systemic inflammation and disease activity, baseline chemokine levels cannot forecast future disease flares over a median follow-up of 5.5 years.

CXCL10 level showed the strongest association with clinical parameters, correlating positively with the SLEDAI score and anti-dsDNA antibodies titers, and inversely with lymphocyte counts. With CXCL10 being an interferon-inducible chemokine, our findings align with the well-established role of the IFN signature in SLE pathogenesis [33]. It is worth highlighting that CXCL10 was elevated in both active and quiescent SLE compared with controls, suggesting its role as a marker of chronic interferon activation [34,35]. The inverse correlation with lymphocyte count observed in our cohort supports the hypothesis that high CXCL10 levels drive lymphocytes out of the circulation and into target organs, contributing to the lymphopenia characteristic of active SLE [13,36]. Moreover, the association of CXCL10 with autoantibody production and systemic inflammatory markers indicates that this chemokine may integrate both immunological activation and inflammatory burden in SLE, reinforcing its potential utility as a biomarker of global disease activity rather than organ-specific involvement [13,14,19].

Similarly, CXCL8, known also as interleukin (IL)-8, levels were markedly elevated in active SLE and correlated with CRP and proteinuria. As a potent neutrophil chemoattractant, CXCL8 plays a pivotal role in neutrophil recruitment and the subsequent release of neutrophil extracellular trap (NET) formation, a process increasingly recognized as a source of autoantigens in SLE (NETosis) [37]. Importantly, NETs can activate mesangial cells in LN, promote proinflammatory and profibrotic responses, and serve as a source of autoantigens, further fueling the inflammatory cascade [38]. The correlation between CXCL8, C-reactive protein, proteinuria and SLEDAI score suggests that it reflects the immediate inflammatory burden rather than chronic, smoldering autoimmunity [11,12].

In contrast to CXCL10 and CXCL8, serum levels of CCL2 and CCL4 did not differ significantly between SLE patients and controls, nor between active and inactive subgroups. Moreover, only weak correlations were detected between CCL2 or CCL4 and other chemokines, and neither showed consistent associations with global disease activity indices or renal parameters. This finding diverges from several previous studies that reported elevated CCL2 in active SLE, particularly in LN [10,14,36,39]. A plausible explanation for this discrepancy lies in the treatment profile of our cohort, as CCL2 expression is sensitive to immunosuppression [9]. Since most of our patients were receiving hydroxychloroquine and other immunosuppressants, and those with a history of severe disease had received substantial cumulative doses of cyclophosphamide, it is plausible that therapy effectively dampened the monocyte-chemokine axis, masking potential elevations [9].

Interestingly, CCL5 was the only chemokine significantly elevated in patients with inactive disease, compared to controls. While usually associated with T-cell recruitment in active inflammation, persistent CCL5 elevation in remission could indicate subclinical immune dysregulation or a shift toward a tissue-repair profile, though this requires further investigation [6]. However, previous studies have reported increased CCL5 expression in active SLE [14,18]. Fu et al. [18] demonstrated that transcriptional levels of interferon-inducible chemokines, including CCL5, were significantly elevated in patients with active SLE, and that a composite chemokine score correlated positively with SLEDAI and was higher in patients with active LN. Similarly, Dominguez-Gutierrez et al. [14] showed increased circulating CCL5 levels in SLE compared with healthy controls, with a positive association between CCL5 expression, interferon activation, and disease activity. In contrast to these observations, in our cohort serum CCL5 concentrations did not differ significantly between patients with active SLE and those in remission or healthy controls.

Although chemokines are major mediators of renal injury, we did not observe significant differences in serum chemokine levels between patients with renal flares and non-renal flares, nor based on a history of nephritis in remission. While this may suggest that serum levels do not adequately reflect local intra-renal production, a known limitation of systemic biomarkers, it is also likely attributable to the limited sample size in the subgroup analysis (n = 8 vs. n = 7). Consequently, while serum CXCL10 and CXCL8 reflect systemic activity, they may lack the specificity required to distinguish renal from non-renal involvement without concurrent urinary analysis. Consistent with previous reports indicating that urinary rather than serum chemokine levels more accurately reflect renal involvement in SLE [36,40], we did not observe significant differences in circulating CCL2, CCL4, CCL5, CXCL8, or CXCL10 between patients with renal and non-renal flares or between remission subgroups, underscoring the limited sensitivity of serum chemokines for discriminating LN.

Another issue that merits comment is whether baseline chemokine levels could predict future disease flares. Despite the clear association with concurrent activity, baseline concentrations failed to predict disease flares in patients followed for over five years. This negative finding is clinically significant. It suggests that chemokine levels are highly dynamic, reflecting the “here and now” of the inflammatory milieu rather than a setpoint of future risk [17,41]. This aligns with the “gap” in evidence regarding longitudinal utility; while these markers are excellent for monitoring current status, a single snapshot measurement during remission is insufficient for long-term risk stratification. Future strategies likely require serial monitoring to detect the “rising tide” of chemokines preceding a flare, rather than relying on a static baseline value.

The primary strength of this study lies in its longitudinal design with a substantial follow-up period (median of 5.5 years), which addresses a significant gap in the current literature. While many previous studies have relied on cross-sectional analyses to associate chemokine levels with concurrent disease activity, our study evaluates the long-term prognostic utility of these markers in a real-world clinical setting. Additionally, the use of the rigorous 2019 EULAR/ACR classification criteria ensures a well-characterized patient cohort. By utilizing the Luminex multiplex assay, we were able to simultaneously measure a panel of five distinct chemokines, providing a comprehensive profile of the inflammatory milieu rather than assessing single mediators in isolation. Our findings must be interpreted considering several limitations. The relatively small sample size (n = 52) and the single-center nature of the study may limit the generalizability of our results to broader SLE populations and reduce statistical power, particularly for subgroup analyses of specific organ manifestations. The relatively small size of the control group should be considered a limitation of this study and may have reduced the statistical power to detect modest differences in demographic and laboratory parameters between controls and patients. A key limitation of this study is the small sample size of the renal flare subgroup (n = 8 vs. n = 7), which substantially limits statistical power and precludes definitive conclusions regarding the absence of differences between groups; therefore, these findings should be interpreted as exploratory rather than confirmatory. Another limitation is the fact that immunosuppressive therapy used by patients could influence chemokine levels, thus our results should be interpreted with caution. Next, estimated glomerular filtration rate was calculated using the MDRD equation, and although the use of the CKD-EPI formula might be more appropriate in cohorts including individuals with preserved renal function, this is unlikely to have materially affected the results and is acknowledged as a study limitation. Furthermore, chemokine concentrations were measured only at baseline. Given the fluctuating nature of SLE and the short half-life of many cytokines, a singular measurement may not fully capture the dynamic inflammatory processes leading up to a flare; serial measurements could potentially yield different predictive insights. Finally, as is common in observational SLE studies, most patients were receiving standard immunosuppressive therapy, which may have modulated serum chemokine levels and attenuated correlations with disease activity.

## 5. Conclusions

Our findings demonstrate that serum CXCL8 and CXCL10 levels are closely associated with disease activity and systemic inflammation in SLE, supporting their utility as biomarkers for monitoring ongoing immune activation. Nevertheless, baseline chemokine concentrations did not predict subsequent disease flares. Our findings support the use of chemokines as complementary tools for monitoring current disease status but caution against their use as standalone prognostic markers for long-term flare prediction. Further studies are needed to assess if CXCL8 or CXCL10 could be treat-to-target chemokines to guide personalized disease management, e.g., maintenance immunosuppressant treatment, and if serial chemokine measurements at set intervals can predict disease flares.

## Figures and Tables

**Table 1:** Demographic and clinical profile of all participants included in the study.

Parameter	All SLE Patients n = 52	Active SLE Patients n = 15	Inactive SLE Patients n = 37	Controls n = 12	*p*-Value All SLE vs. Controls	*p*-Value Active SLE vs. Controls	*p*-Value Inactive SLE vs. Controls	*p*-Value Active SLE vs. Inactive
**Demographic characteristics**								
Age at enrolment, years	49.0 (36.0–62.5)	42.0 (35.0–51.0)	51.0 (36.5–66.0)	37.5 (34.5–44.5)	0.11	>0.99	0.14	0.27
Sex, female, n (%)	46 (88.5%)	14 (93.3%)	32 (86.5%)	8 (66.7%)	0.08	0.14	0.20	0.66
Age at SLE onset, years	29.0 (20.0–43.0)	26.0 (22.0–43.0)	30.0 (20.0–42.5)	NA	NA	NA	NA	0.84
SLE duration, years	17.0 (8.3–22.8)	12.0 (1.0–20.0)	17.0 (10.0–23.0)	NA	NA	NA	NA	0.15
**Symptoms ever stated**								
Skin, n (%)	37 (71.2%)	11 (73.3%)	26 (70.3%)	NA	NA	NA	NA	>0.99
Oral ulceration, n (%)	5 (9.6%)	1 (6.7%	4 (10.8%)	NA	NA	NA	NA	>0.99
Photosensitivity, n (%)	18 (28.1%)	6 (40.0%)	12 (32.4%)	NA	NA	NA	NA	0.60
Joints, n (%)	41 (78.8%)	11 (73.3%)	30 (81.1%)	NA	NA	NA	NA	0.71
Serositis, n (%)	12 (23.1%)	4 (26.7%)	8 (21.6%)	NA	NA	NA	NA	0.73
Renal, n (%)	24 (46.2%)	8 (53.3%)	16 (43.2%)	NA	NA	NA	NA	0.51
Neurologic, n (%)	3 (5.8%)	0 (0.0%)	3 (8.1%)	NA	NA	NA	NA	0.55
Hematologic, n (%)	47 (90.4%)	14 (93.1%)	33 (89.2%)	NA	NA	NA	NA	>0.99
**Symptoms at the study enrolment**								
Skin, n (%)	7 (13.5%)	6 (40.0%)	1 (2.7%)	NA	NA	NA	NA	**0.001**
Joints, n (%)	9 (17.3%)	9 (60.0%)	0 (0.0%)	NA	NA	NA	NA	**<0.001**
Serositis, n (%)	3 (5.8%)	3 (20.0%)	0 (0.0%)	NA	NA	NA	NA	**0.021**
Renal, n (%)	8 (15.4%)	8 (53.3%)	0 (0.0%)	NA	NA	NA	NA	**<0.001**
Neurologic, n (%)	0 (0.0%)	0 (0.0%)	0 (0.0%)	NA	NA	NA	NA	NA
Hematologic, n (%)	18 (34.6%)	10 (66.7%)	8 (21.6%)	NA	NA	NA	NA	**0.002**
**Clinical and laboratory characteristics**								
SLEDAI, score	2.0 (0.0–7.5)	12.0 (10.0–21.0)	2.0 (0.0–3.0)	NA	NA	NA	NA	**<0.001**
rSLEDAI, score	0.0 (0.0–0.0)	8.0 (0.0–12.0)	0.0 (0.0–0.0)	NA	NA	NA	NA	**<0.001**
ANA, titer	1:5120 (1:160–1:20,480)	1:5120 (1:320–1:20,480)	1:2560 (1:160–1:20,480)	1:640 (1:320–1:2560)	**0.008**	**0.003**	0.10	0.09
Anti-dsDNA antibodies, titer	<1:10 (<1:10–1:10,240)	1:40 (<1:10–1:10,240)	<1:10 (<1:10–1:640)	<1:10 (<1:10-<1:10)	0.09	**0.017**	0.49	**0.037**
Proteinuria, g/day	0.13 (0.09–0.44)	0.52 (0.14–1.79)	0.11 (0.08–0.17)	0.08 (0.04–0.12)	0.06	**0.007**	0.57	**0.006**
Creatinine, µmol/L	61.5 (56.3–76.5)	60.0 (50.0–93.0)	62.0 (57.0–74.0	77.0 (60.5–83.5)	0.31	0.92	0.96	>0.99
eGFR, ml/min/1.73 m^2^	98.0 (77.0–109.5)	100.0 (59.0–138.0)	98.0 (77.0–106.5)	97.0 (80.0–110.5)	0.99	>0.99	>0.99	>0.99
C3c, g/L	1.01 (0.80–1.24)	0.81 (0.63–1.16)	1.03 (0.85–1.27)	0.95 (0.73–1.24)	0.75	>0.99	>0.99	0.12
C4, g/L	0.15 (0.10–0.20)	0.08 (0.07–0.17)	0.17 (0.13–0.20)	0.20 (0.18–0.23)	0.06	**0.010**	0.52	**0.014**
C-reactive protein, mg/L	2.3 (1.0–5.1)	5.1 (2.1–21.0)	1.8 (1.0–3.6)	1.0 (1.0–1.6)	**0.046**	**0.018**	0.37	0.08
White blood cells, 10^3^/µL	5.0 (3.8–7.5)	4.6 (2.5–5.6)	5.3 (4.0–8.2)	4.7 (4.0–6.3)	0.84	>0.99	>0.99	0.33
Lymphocytes, 10^3^/µL	1.2 (0.8–1.7)	0.8 (0.6–1.2)	1.4 (0.9–1.8)	1.8 (1.2–2.1)	0.13	**0.042**	0.86	**0.038**
Neutrophils, 10^3^/µL	3.0 (2.3–5.3)	3.2 (1.5–5.1)	2.8 (2.3–5.4)	2.4 (2.3–3.6)	0.56	>0.99	>0.99	0.83
Platelets, 10^3^/µL	242 (194–277)	246 (134–286)	240 (196–272)	258 (201–321)	0.63	>0.99	>0.99	>0.99
Current sGCS, mg/day	0.0 (0.0–4.0)	0.0 (0.0–4.0)	0.0 (0.0–4.0)	NA	NA	NA	NA	0.41
Cumulative CTX dose, g	0.0 (0.0–10.0)	0.0 (0.0–10.0)	0.0 (0.0–11.8)	NA	NA	NA	NA	0.95

Categorical variables are presented as numbers with percentages. Continuous variables are presented as medians with Q1–Q3 ranges (medians with min-max range for ANA and anti-dsDNA titers). Statistically significant differences are marked in bold. Statistically significant differences are bolded. Abbreviations: ANA—anti-nuclear antibodies, C3c—complement component 3, C4—complement component 4, CTX—cyclophosphamide, eGFR—estimated Glomerular Filtration Rate, n—number, NA—not applicable, rSLEDAI—renal SLEDAI, sGCS—systemic glucocorticosteroids (recounted for methylprednisolone), SLE—systemic lupus erythematosus, SLEDAI—SLE disease activity index.

**Table 2:** Levels of selected chemokines in active and inactive systemic lupus erythematosus patients and controls.

Selected Chemokine	SLE Patients n = 52	Active SLE Patients n = 15	Inactive SLE Patients n = 37	Controls n = 12	*p*-Value All SLE vs. Controls	*p*-Value Active SLE vs. Controls	*p*-Value Inactive SLE vs. Controls	*p*-Value Active SLE vs. Inactive SLE
CCL2, pg/mL	432.1 (348.3–659.3)	401.4 (353.8–1112.6)	436.4 (337.2–611.3)	441.2 (258.5–539.6)	0.43	>0.99	>0.99	>0.99
CCL4, pg/mL	261.3 (194.5–345.3)	317.4 (194.5–429.2)	261.3 (194.5–306.8)	236.0 (194.5–249.0)	0.10	0.12	0.64	0.62
CCL5, pg/mL	46,035.4 (28,781.0–57,738.5)	39,978.6 (26,846.1–53,603.8)	48,087.5 (32,111.1–60,410.7)	34,278.1 (20,863,0–42,245.5)	**0.013**	0.44	**0.021**	0.83
CXCL8, pg/mL	14.04 (10.03–25.75)	28.85 (14.17–47.43)	12.92 (8.65–17.91)	10.16 (7.26–24.94)	0.35	**0.049**	>0.99	**0.011**
CXCL10, pg/mL	35.00 (22.26–61.31)	45.35 (32.91–166.21)	32.43 (20.38–54.03)	16.15 (13.93–21.92)	**<0.001**	**<0.001**	**0.018**	0.12

Continuous variables are presented as median with Q1–Q3 ranges. Statistically significant differences are bolded. Abbreviations: CCL5—C-C motif chemokine ligand 5 (RANTES), CCL2—C-C motif chemokine ligand 2 (MCP-1), CCL4—C-C motif chemokine ligand 4 (MIP-1β), CXCL10—C-X-C motif chemokine ligand 10 (IP-10), CXCL8—C-X-C motif chemokine ligand 8 (IL-8), SLE—systemic lupus erythematosus.

**Table 3:** Levels of selected cytokines in active systemic lupus erythematosus patients with renal flare and other than renal flare.

Parameter	Active SLE Patients with Renal Flare n = 8	Active SLE Patients with Non-Renal Flare n = 7	*p*-Value
**Cytokines measurements**			
CCL2, pg/mL	384.63 (288.58–1461.78)	437.09 (353.79–1112.57)	0.61
CCL4, pg/mL	355.96 (225.22–1383.99)	249.03 (194.49–394.53)	0.28
CCL5, pg/mL	43,576.17 (31,121.60–53,443.40)	36,646.23 (22,454.34–53,976.05)	0.54
CXCL8, pg/mL	32.58 (24.20–49.84)	14.17 (9.40–41.24)	0.23
CXCL10, pg/mL	53.54 (26.99–128.77)	44.07 (32.91–171.13)	0.78

Continuous variables are presented as medians with Q1–Q3 ranges. Abbreviations: CCL5—C-C motif chemokine ligand 5 (RANTES), CCL2—C-C motif chemokine ligand 2 (MCP-1), CCL4—C-C motif chemokine ligand 4 (MIP-1β), CXCL10—C-X-C motif chemokine ligand 10 (IP-10), CXCL8—C-X-C motif chemokine ligand 8 (IL-8), SLE—systemic lupus erythematosus.

**Table 4:** Levels of selected cytokines in inactive systemic lupus erythematosus patients with remission and lupus nephritis versus remission without lupus nephritis.

Parameter	Inactive SLE Patients with Remission and Lupus Nephritis n = 16	Inactive SLE Patients with Remission Without Lupus Nephritis n = 21	*p*-Value
**Cytokines measurements**			
CCL2, pg/mL	429.20 (339.78–594.22)	460.81 (305.08–698.74)	0.55
CCL4, pg/mL	236.01 (170.51–369.20)	261.32 (208.74–301.35)	0.53
CCL5, pg/mL	52,845.02 (30,111.77–71,950.31)	47,344.07 (32,111.06–58,001.15)	0.51
CXCL8, pg/mL	12.42 (7.26–22.33)	12.92 (9.40–16.17)	0.89
CXCL10, pg/mL	30.70 (17.90–55.37)	32.43 (21.72–54.03)	0.42

Continuous variables are presented as medians with Q1–Q3 ranges. Abbreviations: CCL5—C-C motif chemokine ligand 5 (RANTES), CCL2—C-C motif chemokine ligand 2 (MCP-1), CCL4—C-C motif chemokine ligand 4 (MIP-1β), CXCL10—C-X-C motif chemokine ligand 10 (IP-10), CXCL8—C-X-C motif chemokine ligand 8 (IL-8), SLE—systemic lupus erythematosus.

**Table 5:** Association of selected cytokines with clinical and laboratory parameters in all studied patients with systemic lupus erythematosus.

Parameter	CCL2, pg/mL	CCL4, pg/mL	CCL5, pg/mL	CXCL8, pg/mL	CXCL10, pg/mL
Age at enrolment, years	r_S_ = 0.15, *p* = 0.20	r_S_ = 0.11, *p* = 0.42	r_S_ = −0.02, *p* = 0.90	r_S_ = −0.16, *p* = 0.26	r_S_ = 0.09, *p* = 0.52
Age at SLE onset, years	r_S_ = 0.04, *p* = 0.77	r_S_ = 0.11, *p* = 0.43	r_S_ = 0.03, *p* = 0.84	r_S_ = −0.03, *p* = 0.83	r_S_ = 0.06, *p* = 0.65
SLE duration, years	r_S_ = 0.04, *p* = 0.78	r_S_ = −0.04, *p* = 0.77	r_S_ = 0.10, *p* = 0.49	r_S_ = −0.09, *p* = 0.52	r_S_ = −0.07, *p* = 0.61
SLEDAI, score	r_S_ = 0.08, *p* = 0.57	r_S_ = 0.25, *p* = 0.07	r_S_ = −0.06, *p* = 0.66	**r_S_ = 0.44,** ***p* = 0.001**	**r_S_ = 0.37,** ***p* = 0.007**
rSLEDAI, score	r_S_ = 0.00, *p* = 0.98	r_S_ = 0.17, *p* = 0.22	r_S_ = 0.04, *p* = 0.78	**r_S_ = 0.42,** ***p* = 0.002**	r_S_ = 0.12, *p* = 0.39
ANA, titer	r_S_ = 0.06, *p* = 0.68	r_S_ = 0.15, *p* = 0.28	r_S_ = −0.12, *p* = 0.40	r_S_ = 0.17, *p* = 0.24	**r_S_ = 0.35,** ***p* = 0.011**
Anti-dsDNA antibodies, titer	**r_S_ = 0.38,** ***p* = 0.005**	r_S_ = 0.22, *p* = 0.12	r_S_ = −0.79, *p* = 0.49	**r_S_ = 0.32,** ***p* = 0.021**	**r_S_ = 0.29,** ***p* = 0.036**
Proteinuria, g/day	r_S_ = −0.04, *p* = 0.78	r_S_ = 0.16, *p* = 0.27	r_S_ = −0.04, *p* = 0.77	**r_S_ = 0.29,** ***p* = 0.040**	r_S_ = 0.01, *p* = 0.93
Creatinine, µmol/L	r_S_ = −0.20, *p* = 0.16	r_S_ = 0.01, *p* = 0.97	r_S_ = −0.16, *p* = 0.27	r_S_ = −0.02, *p* = 0.91	r_S_ = −0.01, *p* = 0.49
eGFR, ml/min/1.73 m^2^	r_S_ = 0.12, *p* = 0.41	r_S_ = −0.02, *p* = 0.89	r_S_ = 0.15, *p* = 0.30	r_S_ = 0.08, *p* = 0.60	r_S_ = 0.07, *p* = 0.60
C3c, g/L	r_S_ = 0.08, *p* = 0.59	r_S_ = −0.26, *p* = 0.06	**r_S_ = 0.36,** ***p* = 0.008**	r_S_ = −0.08, *p* = 0.57	r_S_ = −0.22, *p* = 0.12
C4, g/L	r_S_ = −0.12, *p* = 0.41	**r_S_ = −0.34,** ***p* = 0.013**	**r_S_ = 0.38,** ***p* = 0.006**	**r_S_ = −0.32,** ***p* = 0.020**	**r_S_ = −0.32,** ***p* = 0.020**
C-reactive protein, mg/L	**r_S_ = 0.31,** ***p* = 0.027**	**r_S_ = 0.29,** ***p* = 0.043**	r_S_ = −0.19, *p* = 0.18	**r_S_ = 0.42,** ***p* = 0.003**	**r_S_ = 0.45, *p* < 0.001**
White blood cells, 10^3^/µL	r_S_ = 0.05, *p* = 0.75	r_S_ = −0.19, *p* = 0.18	r_S_ = 0.20, *p* = 0.16	r_S_ = −0.05, *p* = 0.71	**r_S_ = −0.34,** ***p* = 0.013**
Lymphocytes, 10^3^/µL	r_S_ = −0.03, *p* = 0.82	r_S_ = −0.09, *p* = 0.55	r_S_ = 0.14, *p* = 0.32	r_S_ = −0.13, *p* = 0.34	**r_S_ = −0.36,** ***p* = 0.008**
Neutrophils, 10^3^/µL	r_S_ = 0.00, *p* = 0.98	r_S_ = −0.22, *p* = 0.12	r_S_ = 0.19, *p* = 0.18	r_S_ = −0.07, *p* = 0.62	**r_S_ = −0.32,** ***p* = 0.019**
Platelets, 10^3^/µL	r_S_ = 0.04, *p* = 0.80	r_S_ = 0.05, *p* = 0.73	**r_S_ = 0.47, *p* < 0.001**	r_S_ = 0.06, *p* = 0.66	r_S_ = −0.19, *p* = 0.18
Current sGCS, mg/day	r_S_ = 0.03, *p* = 0.82	**r_S_ = −0.29,** ***p* = 0.040**	r_S_ = −0.09, *p* = 0.54	r_S_ = −0.20, *p* = 0.16	r_S_ = −0.14, *p* = 0.33
Cumulative CTX dose, g	r_S_ = −0.04, *p* = 0.78	r_S_ = −0.14, *p* = 0.32	r_S_ = 0.19, *p* = 0.18	r_S_ = 0.00, *p* > 0.99	r_S_ = −0.17, *p* = 0.22

Correlation matrix showing significant associations between selected cytokines and parameters related to demographics and clinical data in the whole SLE group. Statistically significant differences are bolded. Statistically significant differences are bolded. Abbreviations: ANA—antinuclear antibodies, C—complement component, CTX—cyclophosphamide (cumulative dose), n—number, rSLEDAI—renal SLEDAI, SLE—systemic lupus erythematosus, SLEDAI—SLE Disease Activity Index, sGCS—systemic glucocorticoids (recounted for methylprednisolone), eGFR—estimated Glomerular Filtration Rate, n—number, CCL5—C-C motif chemokine ligand 5 (RANTES), CCL2—C-C motif chemokine ligand 2 (MCP-1), CCL4—C-C motif chemokine ligand 4 (MIP-1β), CXCL10—C-X-C motif chemokine ligand 10 (IP-10), CXCL8—C-X-C motif chemokine ligand 8 (IL-8), SLE—systemic lupus erythematosus.

**Table 6:** Levels of selected cytokines in inactive systemic lupus erythematosus patients with a flare and without a flare in the follow-up analysis.

Parameter	Inactive SLE Patients with a Flare in Follow-Up n = 10	Inactive SLE Patients Without a Flare in Follow-Up n = 27	*p*-Value
**Cytokines measurements**			
CCL2, pg/mL	409.44 (348.94–693.25)	460.81 (327.92–613.88)	0.83
CCL4, pg/mL	278.61 (153.15–324.18)	249.03 (194.49–306.79)	0.80
CCL5, pg/mL	45,955.94 (25,244.41–56,754.98)	50,930.84 (37,826.16–65,897.56)	0.37
CXCL8, pg/mL	12.67 (7.64–14.29)	13.17 (9.91–19.16)	0.41
CXCL10, pg/mL	35.62 (18.98–75.71)	32.43 (20.30–47.26)	0.67

Continuous variables are presented as medians with Q1–Q3 ranges. Abbreviations: CCL5—C-C motif chemokine ligand 5 (RANTES), CCL2—C-C motif chemokine ligand 2 (MCP-1), CCL4—C-C motif chemokine ligand 4 (MIP-1β), CXCL10—C-X-C motif chemokine ligand 10 (IP-10), CXCL8—C-X-C motif chemokine ligand 8 (IL-8), SLE—systemic lupus erythematosus, n—number.

## Data Availability

The data presented in this study are available on request from the corresponding author. The data are not publicly available due to privacy and ethical restrictions.
